# Anticodon-engineered tRNAs restore full-length MeCP2 expression and function in Rett syndrome nonsense mutations

**DOI:** 10.3389/fneur.2026.1778877

**Published:** 2026-06-01

**Authors:** Elena Fara, Stefano Pezzini, Ginevra Arpaia, Angelisa Frasca, Joseph J. Porter, John D. Lueck, Nicoletta Landsberger

**Affiliations:** 1 Laboratory of Molecular and Cellular Biology Applied to Neurodevelopmental Disorders, Department of Medical Biotechnology and Translational Medicine, University of Milan, Milan, Italy; 2 San Raffaele Rett Research Unit, Neuroscience Division, IRCCS San Raffaele Scientific Institute, Milan, Italy; 3 Laboratory of Experimental Neurobiology, Department of Medical Biotechnology and Translational Medicine, University of Milan, Milan, Italy; 4 Department of Pharmacology and Physiology, School of Medicine and Dentistry, University of Rochester, Rochester, NY, United States; 5 Department of Neurology, School of Medicine and Dentistry, University of Rochester, Rochester, NY, United States; 6 Center for RNA Biology, School of Medicine and Dentistry, University of Rochester, Rochester, NY, United States

**Keywords:** ACE-tRNAs, MeCP2, nonsense mutations, readthrough, Rett syndrome

## Abstract

**Background:**

Rett syndrome (RTT) is a severe neurodevelopmental disorder most commonly caused by loss-of-function mutations in the *MECP2* gene, including a substantial fraction of nonsense variants. Despite intense research efforts, no curative therapy is currently available for RTT, although several therapeutic strategies are under investigation. Among these, translational readthrough approaches, aimed at promoting ribosomal bypass of premature termination codons (PTCs) using small molecules, have attracted considerable attention in recent years. However, chemical readthrough agents raise concerns due to their limited specificity and the high sensitivity of MeCP2 to amino acid substitutions, underscoring the need for strategies capable of restoring full-length MeCP2 while preserving its amino acidic and functional integrity.

**Methods:**

Using cellular models expressing common RTT-associated nonsense variants of *MECP2*, we investigated the potential of anticodon-edited transfer RNAs (ACE-tRNAs) to selectively suppress *MECP2* nonsense mutations and restore full-length, functional MeCP2 protein.

**Results:**

We show that ACE-tRNAs promote efficient and dose-dependent readthrough, leading to the production of full-length MeCP2. Readthrough efficiency differed among nonsense mutations, with only specific variants benefitting from ACE-tRNA level optimization. Functional analyses revealed that ACE-tRNA–rescued MeCP2 correctly localizes to heterochromatic foci and regains the ability to recruit the transcriptional corepressor TBL1, two hallmark features of MeCP2 activity. In contrast, scrambled control tRNAs failed to restore nuclear localization or protein–protein interactions.

**Conclusion:**

Together, these findings demonstrate that ACE-tRNAs enable efficient and functionally relevant readthrough of *MECP2* nonsense mutations, highlighting mutation-specific requirements and supporting the feasibility of a precision readthrough strategy for RTT. Although further *in vivo* validation and delivery optimization is required, ACE-tRNA-based readthrough represents a promising and complementary strategy for the treatment of nonsense mutations associated with RTT.

## Introduction

Rett syndrome (RTT) is a severe neurodevelopmental disorder that primarily affects females, with an estimated prevalence of approximately 1 in 10,000 live-born girls (1). The disorder typically manifests after an apparently normal developmental period of 6–18 months, with a rapid regression phase during which most previously acquired skills, such as dexterity in hand use and verbal abilities, are lost. This phase is accompanied by the emergence of characteristic clinical features that include repetitive hand stereotypies, ataxia, apraxia, and autism-like behaviors. Following regression, disease progression stabilizes; lost skills are not recovered, and multiple comorbidities arise, including respiratory dysrhythmias, dysphagia, epilepsy, progressive scoliosis, hypotonia, osteoporosis, gastrointestinal dysfunctions and behavioral abnormalities (1, 2).

The majority of RTT cases are caused by *de novo* mutations in the paternally derived X-linked *MECP2* allele (3), which encodes methyl-CpG-binding protein 2 (MeCP2). MeCP2 is a multifunctional protein that plays a key role in the regulation of gene expression in the brain (4). MeCP2 comprises three principal functional domains: an N-terminal methyl-CpG-binding domain (MBD), which recognizes methylated cytosines in both CG and CH contexts; a transcriptional repression domain (TRD) which mediates transcriptional repression mainly through the recruitment of NCoR/SMRT corepressor complexes containing histone deacetylase activity; and a C-terminal domain that modulates MeCP2 interaction with DNA and chromatin (5, 6). A nuclear localization signal is located within the TRD. To date, more than 600 pathogenic variants associated with RTT have been identified. These are predominantly loss-of-function mutations and include missense, nonsense, frameshift and deletion variants distributed throughout the coding sequence (1, 7, 8). Notably, eight recurrent hotspot mutations (R106W, R133C, T158M, R168X, R255X, R270X, R294X and R306C) collectively account for approximately 65% of RTT cases (1). Importantly, nonsense mutations represent a clinically relevant fraction of RTT-associated *MECP2* variants and are frequently associated with severe phenotypes, making them a target of interest for therapeutic development (9, 10).

Although RTT was shown nearly two decades ago to be a reversible condition in mouse models (11), no curative treatment is currently available for patients. However, a first drug based on a long-acting IGF1-derived tripeptide has recently received regulatory approval in the United States and Canada, and several additional clinical trials are ongoing or expected to begin soon (12, 13). Among these, preclinical evidence supports gene therapy as a promising strategy. However, its high cost, together with the likelihood that, at least in the near term, treated RTT patients will continue to require adjunctive interventions, underscore the need for complementary and economically accessible strategies beyond gene therapy.

In this context, translational readthrough approaches, that promote ribosomal bypass of premature termination codons (PTCs) appear particularly appealing (9, 10). Several readthrough strategies have been developed over the years, including small molecules such as gentamicin and geneticin, which bind the ribosomal decoding center, alter translation fidelity, and allow the incorporation of near-cognate amino acids at PTC sites (14, 15). In contrast, Ataluren works through a distinct mechanism, whereby it binds multiple sites of the ribosome and competes for release factor binding (16). The efficacy of these compounds on common RTT-associated nonsense mutations has been investigated both *in vitro*, in human and mouse cells, and *in vivo*, in mouse models of the disease. These studies demonstrated restoration of full-length MeCP2 protein synthesis, albeit with variable efficiency depending on the specific stop codon, its surrounding nucleotide context, and the position of the PTC within the *MECP2* transcript (10, 17–19). However, *in vivo* evidence of therapeutic efficacy remains limited. Moreover, these compounds frequently promote the incorporation of incorrect amino acids at the PTC site, raising concerns about their suitability for the treatment of *MECP2* nonsense mutations. Indeed, the large number of pathogenic missense variants distributed throughout the entire protein suggests that MeCP2 is sensitive to sequence perturbations.

Together, these limitations highlight a significant unmet need for readthrough strategies that ensure both efficient suppression of nonsense mutations and accurate restoration of a functional, wild-type protein. Anticodon-edited tRNAs (ACE-tRNAs) offer an attractive alternative strategy for translational readthrough therapy (20, 21). ACE-tRNAs are generated from endogenous human tRNAs by editing their anticodons to recognize one of the three stop codons (UGA, UAA or UAG). Because critical identity elements required for recognition by endogenous aminoacyl-tRNA synthetases are preserved, ACE-tRNAs are efficiently charged with their cognate amino acid and can participate in translational elongation at the targeted PTC, resulting in the production of a full-length, wild-type protein (20, 22, 23).

ACE-tRNAs have not yet been evaluated for *MECP2* nonsense mutations. This knowledge gap prompted us to investigate the potential of ACE-tRNAs to suppress common RTT-associated PTCs and restore MeCP2 subnuclear distribution that reflects correct chromatin association, and its ability to interact with key molecular partners.

## Materials and methods

### Plasmids

The plasmid pGFP-hMeCP2, containing the WT hMeCP2 cDNA has been generated and described in Marchi et al. (24). Nonsense variants R168X, R255X and R270X fused at the N-terminus with GFP were generated by site-directed mutagenesis. DNA sequence was verified by Sanger automated sequencing. The corresponding GFP-empty plasmid was used as control. Constructs carrying one (1X) or four (4X) copies of the arginine UGA-suppressing ACE-tRNA transgene, as well as scrambled (SCR) tRNA controls, are described in Ref. (23), Supplementary Figure 3. The parental sequence used for suppressor design was tRNA-Arg-TCT-3-1 (tRNA-Arg-TCT-3-1 → UCA).

**tRNA-Arg-UCA-3-1**: **AGCGCTCCGGTTTTTCTGTGCTGAACC TCAGGGGACGCCGACACACGT ACACGTC** *GGCTCTGTGGCGCAATGGATAGCGCATTGGA CTTCAAATTCAAAGGTTGTGGGTTCGAGTCCCACCAGAGTCG* ***GTCC* TTTTTTT**. The 55-bp 5′ leader (also called upstream control element or UCE) is shown in bold, the ACE-tRNA sequence is shown in italics, the anticodon sequence is underlined, the 4-bp 3′ trailer is shown in bold italics, and the RNA pol. III terminator sequence (7Ts) is shown as underlined bold sequence.

The parental sequence used for suppressor design was tRNA-Arg-TCT-3-1 (tRNA-Arg-TCT-3-1 → UCA). The TBL1-mCherry construct (6) was generously supplied by Dr. Adrian Bird (Wellcome Trust Centre for Cell Biology, University of Edinburgh, UK).

### Cell cultures and transfection

HEK293T cells were maintained in DMEM (#D5671, Merck) supplemented with 10% FBS (#F0804, Life Technologies), 1% L-glutamine (#G7513, Merck), and 1% penicillin/streptomycin (#P0781, Merck) at 37 °C in 5% CO_2_. At confluence, cells were detached by gentle pipetting and seeded in 24-well plates for Western blot analyses.

NIH3T3 fibroblasts were cultured under identical conditions, rinsed with D-PBS (#ECB4004AL, Euroclone), detached with trypsin–EDTA (#T4174, Sigma–Merck), and plated onto coverslips in 24-well plates for immunofluorescence.

HEK293T cells were transfected at 70–80% confluence with a total of 0.8 μg of DNA using the calcium phosphate method. Ratios of GFP-MeCP2 to ACE-tRNA constructs (1X, 4X, or SCR) ranged from 1:1 to 1:7. NIH3T3 fibroblasts at similar confluence were transfected with 0.8 or 1.0 μg DNA using Lipofectamine 2000 (#11668027, Thermo Fisher Scientific) at a 1:1 ratio (GFP-MeCP2:ACE-tRNA), or at a ratio 3:3:1 (GFP-MeCP2 WT or variants:ACE-tRNA:TBL1-mCherry). Proper nuclear localization and functional activity of recoded MeCP2 were assessed 24 h post-transfection by fluorescence microscopy.

### Protein extraction and Western blot

HEK293T cells were lysed in RIPA buffer (50 mM Tris–HCl, 150 mM NaCl, 1% NP-40, 0.5% sodium deoxycholate, 0.1% SDS) supplemented with 1X Halt™ Protease and Phosphatase Inhibitor Cocktail (#78444 Thermo Fisher Scientific). Lysates were centrifuged at 14,000×*g* for 10 min, and protein concentration in the resulting supernatant was determined using Pierce™ BCA protein assay kit (#23227 Thermo Fisher scientific). For SDS-PAGE, 20 μg of total protein was mixed with sample buffer (250 mM Tris–HCl pH 6.8, 40% glycerol, 6% SDS), heated at 95 °C for 5 min, and separated on either 10% TGX™ polyacrylamide gels or 4–15% TGX™ precast gels (Bio-Rad). Proteins were transferred onto nitrocellulose membranes using a semi-dry transfer apparatus (Trans-blot SD; Bio-Rad), blocked for 1 h in 5% milk in TBS-T (Tris-buffered saline, 0.1% Tween-20) and incubated overnight at 4 °C with mouse monoclonal anti-GFP antibody (#11814460001, Roche; 1:1,000). HRP-conjugated secondary antibody (1:5000; Jackson ImmunoResearch) was used for detection with *Westar sun* (Cyanagen) or *Clarity Western ECL Substrate* (Bio-Rad) on the Uvitec imaging system (Cleaver Scientific). Band quantification was performed with Uvitec software.

### Immunofluorescence

NIH3T3 cells seeded on coverslips were fixed with 4% paraformaldehyde (#SH30264.0, Euroclone) in PBS for 10 min at room temperature and washed twice with PBS. Cells were permeabilized with 0.2% Triton X-100 (#T8532, Sigma-Aldrich), washed three times with PBS containing 0.2% BSA, and counterstained with DAPI (#62248, Thermo Fisher Scientific). After three additional washes, coverslips were mounted using Fluoromount-G™ (#00-4958-02, Thermo Fisher Scientific). GFP or mCherry fluorescence was acquired directly.

For each experimental group, images from at least two coverslips were collected using a widefield microscope (Nikon Eclipse Ti2) equipped with a 40× objective. Exposure time was kept constant, and gain was adjusted to prevent pixel saturation. Fiji software was used for image analysis and cell counting. At least 75 cells per condition were analyzed.

Localization rescue was quantified as the percentage of cells displaying GFP-MeCP2 enrichment on heterochromatic foci. Functional rescue was evaluated as the percentage of cells showing mCherry-TBL1 recruitment to the same foci.

### Statistical analysis

Statistical analyses were performed using GraphPad Prism 10. Outliers were detected by the ROUT test (*Q* = 1%) or Grubb’s test (*α* = 0.05). Normality distribution was assessed using the D’Agostino–Pearson and Shapiro–Wilk tests. For comparisons between two groups an unpaired t-test was applied. When comparing more than two groups, statistical significance was assessed using one-way ANOVA followed by Tukey post-hoc test, or the Kruskal-Wallis test followed by Dunn’s multiple comparisons test for nonparametric data. A *p*-value < 0.05 was considered statistically significant.

## Results

### ACE-tRNAs mediate efficient readthrough of pathogenic *MECP2* nonsense variants

The ability of ACE-tRNAs to suppress premature stop codons (PTCs) in the *MECP2* gene was assessed by analyzing three pathogenic variants in which the CGA arginine codon is mutated to UGA. To this end, we selected the nonsense variants R168X, R255X and R270X, which occur at frequencies of approximately 11.5, 8.5, and 9%, respectively, and together account for almost one third of RTT cases (7, 10). Notably, the R168X lies within the intervening domain separating the MBD from the TRD, while R255X and R270X fall within the transcriptional repression domain (TRD) and disrupt the essential NCoR/SMRT interaction domain, spanning residues 269–309 (6). By impairing key functional domains of MeCP2, all three variants are generally associated with a severe phenotype (25).

**Figure 1:**
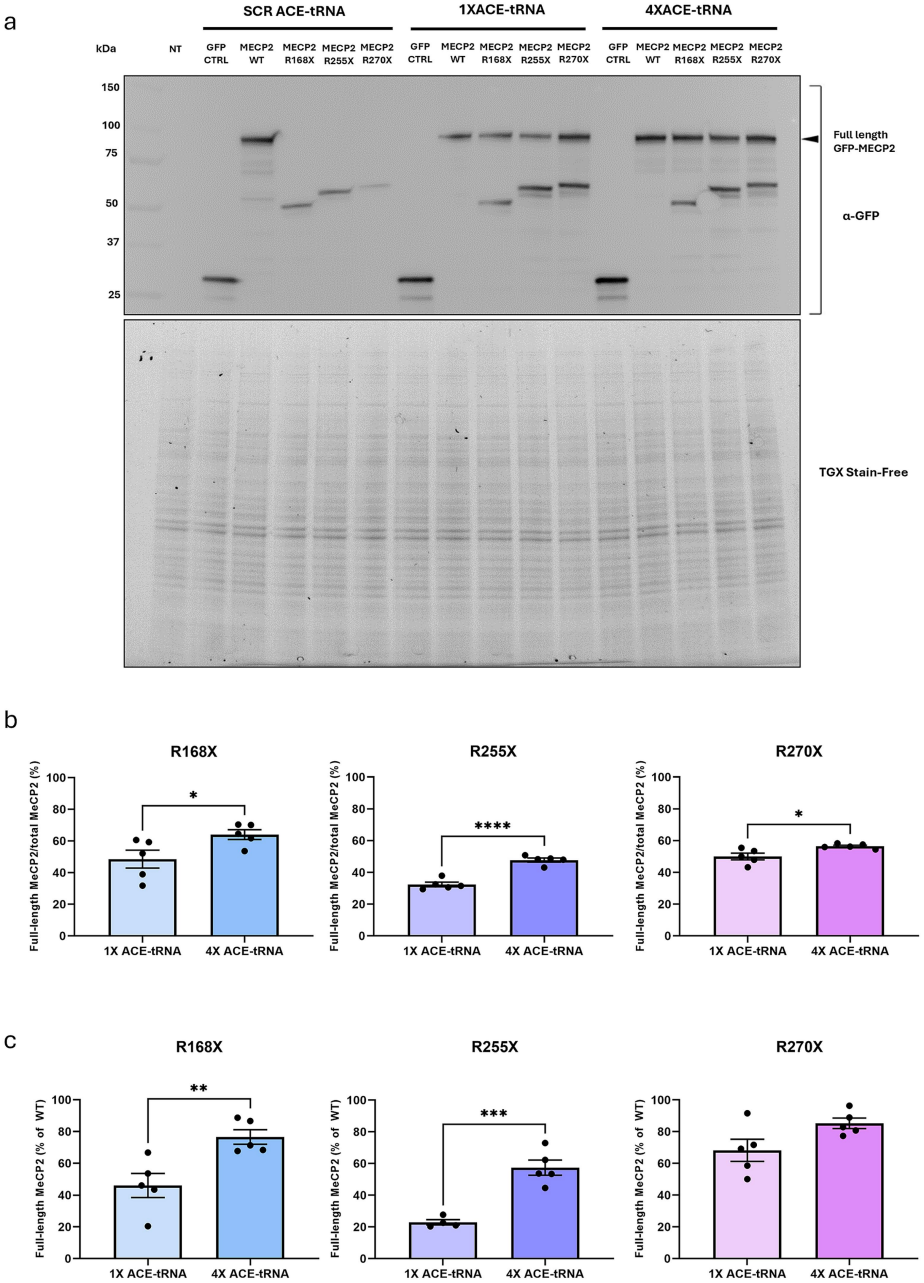
ACE-tRNAs restore full-length MeCP2 protein expression. **(a)** Western blot analysis of GFP control, WT GFP-MeCP2 and the R168X, R255X and R270X nonsense mutants expressed in HEK293T cells and co-transfected with scrambled control (SCR), 1X or 4X ACE-tRNAs. GFP-tagged fusion proteins were detected using an α-GFP antibody. The black arrowhead marks the full-length GFP-MeCP2. Total protein loading was assessed using TGX Stain-Free technology (lower panel). **(b)** Readthrough efficiency was quantified as the ratio of full-length GFP-MeCP2 to the sum of all GFP-MeCP2 bands per lane. **(c)** Readthrough was also quantified by normalizing GFP-MeCP2 signals to the average WT GFP-MeCP2 signals across SCR, 1X and 4X tRNA samples. In panels **(b,c)**, each dot corresponds to an independent biological replicate. Data are expressed as mean ± SEM. *p < 0.05; **p < 0.01; ***p < 0.001; ****p < 0.0001 by unpaired t test.

The selected variants were introduced into a vector expressing full length human MeCP2 cDNA, fused at its N-terminus with GFP (24). To evaluate suppression efficacy, HEK293T cells were transfected with wild-type or mutant GFP-MECP2 constructs together with plasmids encoding either one (1X) or four (4X) copies of ACE-tRNAs. Both vectors were co-transfected in equal amounts (1:1 ratio). Control cells received plasmids encoding a non-functional scramble ACE-tRNA (SCR ACE-tRNA), which lacks suppressor activity and therefore served as a transfection control (23). Comparable transfection efficiency across conditions was revealed by microscopy, by quantifying the percentage of GFP-positive cells. As shown in Figure 1a, western blot analysis demonstrated that both ACE-tRNA constructs restored the synthesis of full-length MeCP2, although to different extents. As previously reported (26), readthrough efficiency was quantified by dividing the intensity of the full-length GFP-MeCP2 band by the sum of truncated and readthrough products. Efficiency varied depending on the specific *MECP2* PTC and on the number of ACE-tRNA copies delivered. With the 1X vector, the R168X and R270X exhibited ~50% recoding, whereas R255X reached only ~32%. Expression of the 4X vector markedly improved readthrough, leading to significantly higher levels of full-length GFP-MeCP2 for all three variants (R168X 64.1%, R255X 47.8% R270X 56.5%) (Figure 1b).

Because the GFP tag might stabilize truncated products, potentially leading to an underestimation of suppression efficiency, and since WT lanes showed MeCP2 levels comparable to those in truncated-plus-4X rescue lanes, we also compared full-length signals from rescued mutants to the average WT GFP-MeCP2 signal obtained across SCR, 1X, and 4X ACE-tRNA co-transfected samples. Consistent with the initial analysis, the 1X ACE-tRNA vector yielded relatively “moderate” rescue, ranging from 46.1% for R168X, to 22.9% for R255X and 68.3% for R270X. In contrast, with the 4X ACE-tRNA construct, recoded MeCP2 reached 76.6% for R168X, 57.4% for R255X, and 85.3% for R270X relative to the WT protein.

Based on these results, aligned with prior observations that increasing the number of ACE-tRNA cassettes enhances suppression efficiency (21, 23, 26, 27), all subsequent experiments were carried out using the 4X ACE-tRNA construct.

### Optimization of transfection ratios enhances MeCP2 readthrough in R255X but not in other variants

**Figure 2:**
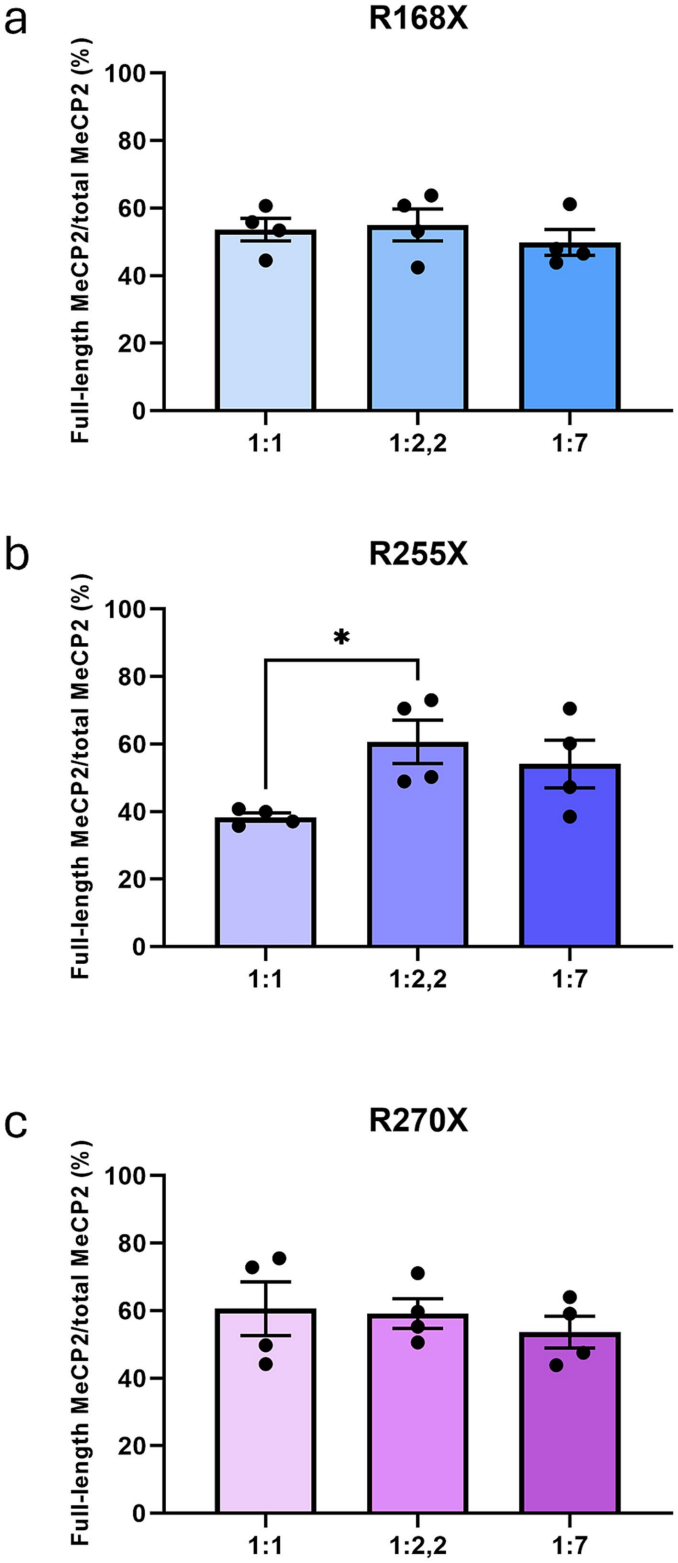
Only the R255X mutation benefits from ACE-tRNA dose optimization. Readthrough efficacy for **(a)** R168X, **(b)** R255X and **(c)** R270X was measured at GFP-MeCP2 to 4X ACE-tRNA transfection ratios of 1:1, 1:2.2, 1:7 and expressed as the ratio of full-length GFP-MeCP2 to the sum of all GFP-MeCP2 bands per lane. In the figure, each dot corresponds to an independent biological replicate. Data are expressed as mean ± SEM. *p < 0.05 by one-way ANOVA followed by Tukey *post hoc* test.

We next assessed whether increasing the amount of ACE-tRNA could improve rescue efficacy. To this end, we evaluated readthrough by delivering different ratios of GFP-MeCP2 to 4X ACE-tRNA, corresponding to 1:1, 1:2.2 and 1:7 (Figure 2). When readthrough was quantified as the proportion of full-length MeCP2 relative to total GFP-MeCP2 products, no improvement was observed for either the R168X or R270X variants at any of the tested ratios (Figures 2a,c).

By contrast, the R255X mutation, which showed the lowest readthrough in the previous experiment, displayed a significant increase in full-length MeCP2 when the plasmid ratio was adjusted from 1:1 (400:400 ng) to 1:2.2 (250:550 ng), reaching nearly 60% rescue (Figure 2b). Delivery of 100:700 ng of vectors resulted in some increase compared to the 1:1 ratio, although this change did not reach statistical significance.

Considering the absence of improvement for R168X and R270X and the limited benefit observed for R255X, we selected the 1:1 plasmid ratio for all the remaining experiments. This ratio ensured robust readthrough, while avoiding unnecessary increase in ACE-tRNA dose, which might support off-target suppression of natural termination codons, an event we previously showed to be undetectable by western blot in these conditions (26) and in other studies (20, 28, 29).

### Recoded MeCP2 properly accumulates at pericentromeric heterochromatin

Proper nuclear localization of MeCP2 can be assessed by examining its accumulation at pericentromeric heterochromatin, which is highly enriched in methylated DNA (30). To determine whether the rescued protein retains this property, we used NIH3T3 murine fibroblasts, which feature well-defined pericentromeric heterochromatic foci and thus provide an ideal model to assess MeCP2 chromatin association (24).

Among the *MECP2* nonsense mutations, we focused on R168X for two main reasons. First, the R168X variant disrupts MeCP2 recognition of methylated DNA and accumulation on heterochromatic foci (31). Second, we have shown efficient readthrough of R168X in HEK293T cells, making it a robust candidate to validate whether suppression restores physiologically correct subcellular localization.

**Figure 3:**
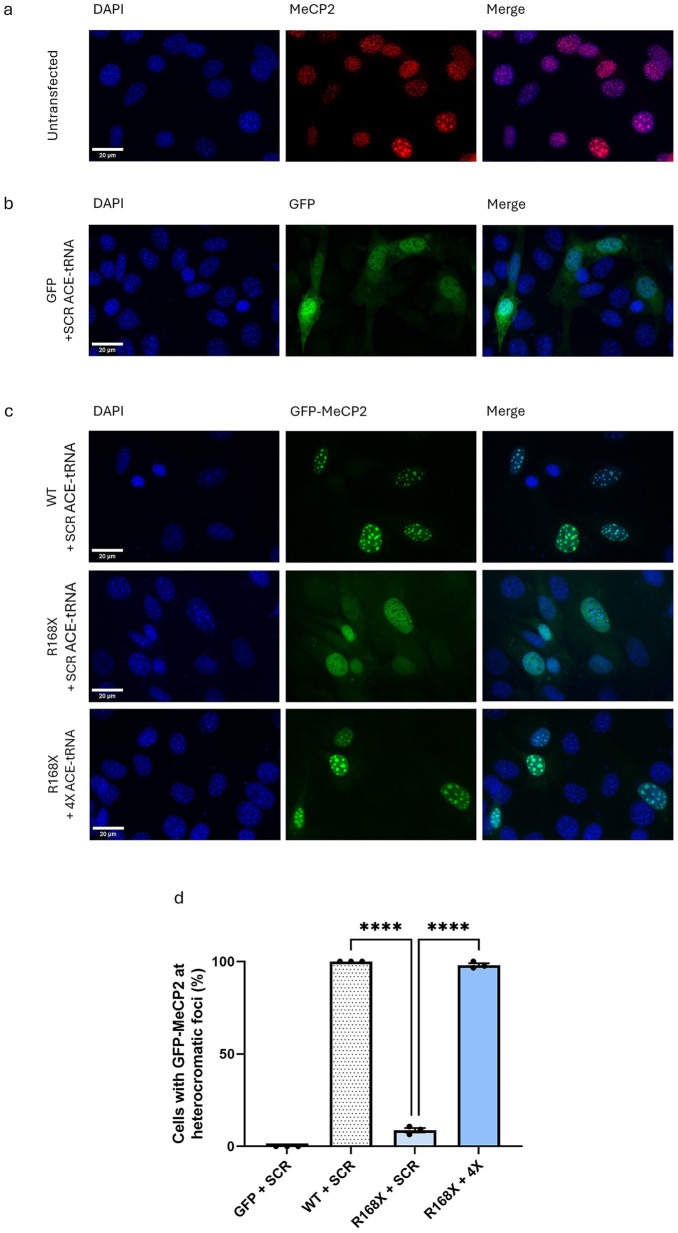
Recoded MeCP2 restores proper localization to heterochromatic foci in NIH3T3 cells. **(a)** Immunofluorescence staining of endogenous MeCP2 in NIH3T3 cells, visualized using DAPI and anti-MeCP2 antibody. **(b)** Representative images of NIH3T3 cells co-transfected with GFP alone (negative control) and SCR tRNA. **(c)** Representative images of NIH3T3 cells co-transfected with WT GFP-MeCP2 or R168X constructs together with SCR or 4X ACE-tRNA. **(d)** The graph shows the percentage of cells displaying clear MeCP2 (green) localization at heterochromatic foci. Each dot represents the mean value obtained from one coverslip derived from an independent experiment (biological *n* = 3). ^****^p < 0.0001; one-way ANOVA followed by Tukey *post hoc* test. For each coverslip, at least four fields containing more than five cells transfected with GFP-MeCP2 or GFP were analyzed. A total of 113, 81, and 121 cells expressing WT GFP-MeCP2 or R168X together with SCR or 4X ACE-tRNA constructs were analyzed for the presence of GFP-MeCP2–positive heterochromatic foci. Values are expressed as percentages of WT + SCR ACE-tRNA, set to 100%, and presented as mean ± SEM. Brightness and contrast adjustments were applied uniformly across all samples to ensure optimal visualization. Scale bar = 20 μm.

Immunofluorescence staining confirmed the expected nuclear pattern for endogenous MeCP2 in NIH3T3 cells, characterized by punctate distribution co-localizing with DAPI-dense heterochromatic foci (Figure 3a). A similar pattern was observed in cells co-transfected with WT GFP-MeCP2 and the SCR-ACE-tRNA, demonstrating correct subnuclear localization (Figure 3c). In contrast, cells transfected with GFP alone showed diffuse fluorescence throughout both the cytoplasm and nucleus, with no enrichment at heterochromatin (Figure 3b).

To assess whether ACE-tRNA–mediated suppression restores proper MeCP2 localization, NIH3T3 cells were co-transfected with R168X GFP-MeCP2 and either SCR or 4X ACE-tRNA at a 1:1 plasmid ratio, based on prior optimization in HEK293T cells. As expected, in the presence of SCR ACE-tRNA, the R168X mutant showed pronounced mislocalization, with fluorescence largely confined to the cytoplasm, minimal nuclear accumulation and detectable heterochromatin-associated foci in only 9% of analyzed transfected cells (Figure 3d).

Remarkably, co-expression of R168X GFP-MeCP2 with 4X ACE-tRNA fully restored nuclear localization, with 98% of analyzed cells displaying punctate accumulation at heterochromatic foci (Figures 3c,d). Notably, in a small fraction of cells, despite correct localization of rescued R168X GFP-MeCP2 to DAPI-positive foci, a diffuse GFP signal was also observed within the nucleus and, in some cases, in the cytoplasm. Altogether, these results indicate efficient suppression of the PTC and restoration of chromatin binding competence, with residual truncated protein persisting in a subset of cells.

### Readthrough MeCP2 restores physiological interaction with NCoR/SMRT

One of the primary functions of MeCP2 is its ability to act as a molecular bridge between the NCoR/SMRT corepressor complexes and chromatin (6). Consistently, a subset of RTT missense mutations disrupts the NCoR/SMRT interaction domain (NID) of MeCP2. Importantly, a peptide spanning residues 285–319 of MeCP2 can directly bind the TBL1 and TBLR1 subunits shared by the two corepressor complexes (6, 32), suggesting that the R168X, R255X and R270X GFP-MeCP2 variants are likely to lose this bridging function.

**Figure 4:**
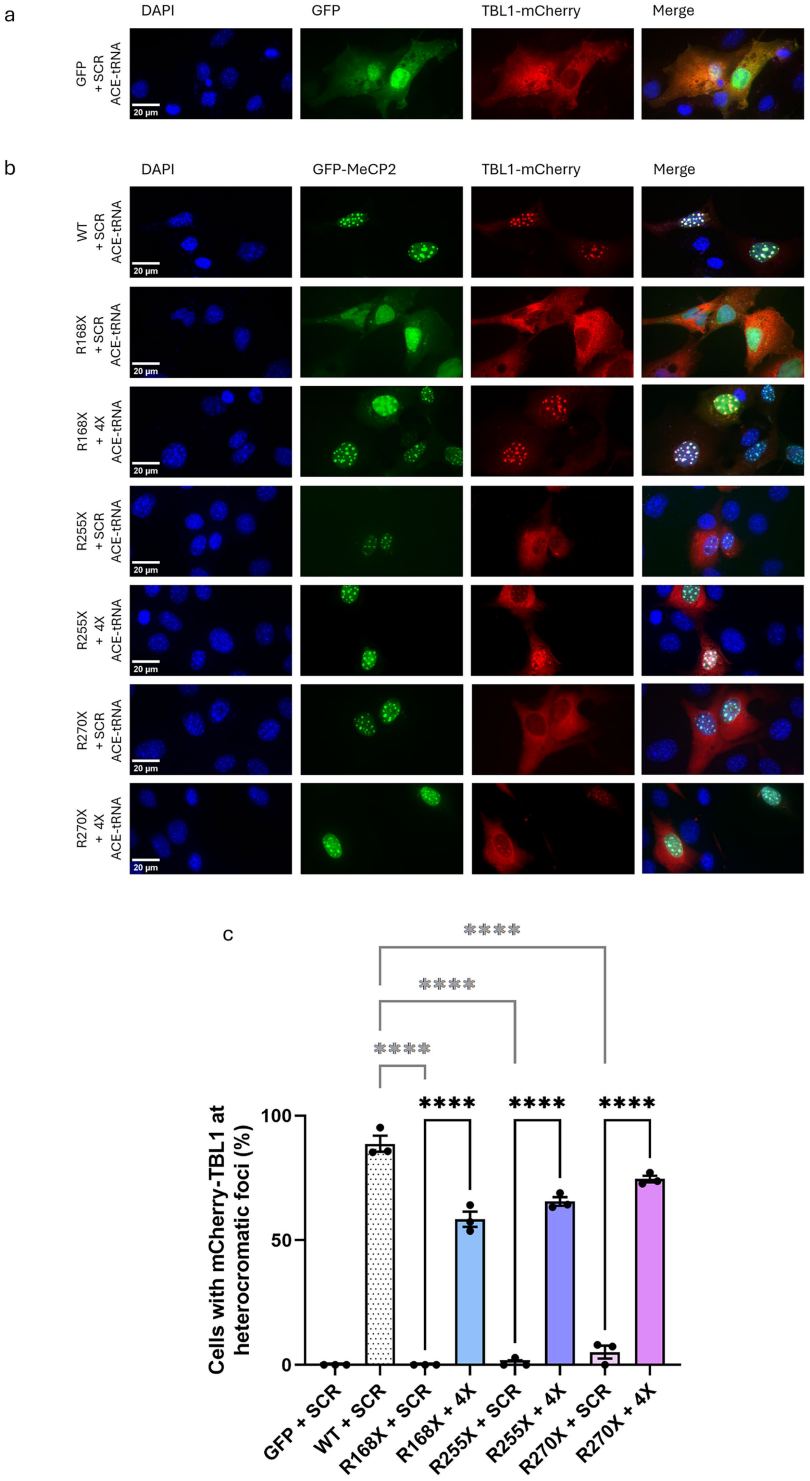
Recoded MeCP2 restores the ability to recruit TBL1 to heterochromatic foci. **(a)** Representative images of NIH3T3 cells co-transfected with TBL1-mCherry, GFP alone and SCR ACE-tRNA (negative control). **(b)** Representative images of cells co-transfected with TBL1-mCherry, and WT or nonsense mutant GFP-MeCP2 constructs together with SCR or 4X ACE-tRNA. **(c)** The graph shows the percentage of cells in which TBL1 (red) was recruited on heterochromatic foci. Each dot represents the mean value obtained from one coverslip derived from an independent experiment (biological *n* = 3). ^****^p < 0.0001; one-way ANOVA followed by Tukey *post hoc* test. For each coverslip, at least four fields containing more than five cells transfected with GFP-MeCP2 or GFP were analyzed. A total of 104, 98, 81, and 75 cells expressing WT GFP–MeCP2, R168X, R255X, or R270X together with TBL1 and SCR ACE-tRNA were analyzed for mCherry–TBL1–positive heterochromatic foci. An identical analysis was performed in 186, 87, and 95 cells expressing R168X, R255X, or R270X, respectively, in the presence of 4X ACE-tRNA. Brightness and contrast adjustments were applied uniformly across samples to ensure optimal visualization. Scale bar = 20 μm.

To determine whether ACE-tRNA-mediated readthrough can rescue this activity, we performed immunofluorescence in NIH3T3 cells. Cells were co-transfected with TBL1-mCherry together with either GFP alone (negative control) (Figure 4a) or with WT GFP-MeCP2 or GFP-MeCP2 carrying the R168X, R255X or R270X PTCs (Figure 4b). For both WT and PTC containing constructs, cells were additionally co-transfected with SCR or 4X ACE-tRNA (Figure 4b). TBL1-mCherry localization was used as a functional readout of NCoR/SMRT recruitment to heterochromatic foci, which strictly depends on the interaction with MeCP2 (6, 33).

NIH3T3 cells expressing GFP alone served as an appropriate negative control, as these cells express insufficient levels of endogenous MeCP2 to recruit significant amounts of exogenously expressed TBL1-mCherry to heterochromatic foci (6, 33). Accordingly, TBL1, which lacks a canonical nuclear localization signal, remained mostly diffuse in the cytoplasm. In contrast, WT GFP-MeCP2 efficiently recruited TBL1-mCherry to pericentromeric heterochromatin, with 89% of analyzed cells displaying bright TBL1-positive puncta overlapping DAPI-dense foci (Figure 4c). This confirms that exogenous MeCP2 correctly bridges NCoR/SMRT complexes on chromatin.

In cells expressing R168X, R255X or R270X GFP-MeCP2 together with SCR tRNA, TBL1-mCherry remained diffuse, with only rare TBL1-positive foci observed (Figure 4b). This pattern confirms that truncated derivatives lacking the NID cannot recruit the NCoR/SMRT complexes to heterochromatin, with accumulation detected in 0% of R168X-, 1% of R255X- and 5% of R270X-expressing cells (Figure 4c). Remarkably, co-expression of 4X ACE-tRNA vectors restored the appearance of TBL1-positive heterochromatic puncta in cells expressing all three nonsense mutants. In a fraction of cells, high levels of TBL1-mCherry were associated with a diffuse cytoplasmic signal, likely reflecting overexpression relative to GFP-MeCP2.

Finally, because recoded GFP-MeCP2 variants suggested some differences in nuclear diffusibility, we objectively assessed this by quantifying the nucleoplasmic-to-total nuclear signal ratio. The analysis showed that all samples display comparable diffusibility, with no measurable effect of ACE-tRNAs. Only the R168X mutant-lacking the methyl-DNA-binding domain-showed a slightly more diffuse distribution (Supplementary Figure S1).

These findings indicate that readthrough-derived full-length MeCP2 is competent to reestablish its interaction with TBL1 and to tether NCoR/SMRT complexes to chromatin, thereby restoring a key aspect of MeCP2 function. Rescue efficiency varied among mutants but remained robust and reproducible, with 59% for R168X-, 66% for R255X, and 75% for R270X-expressing cells. No recruitment was observed in the corresponding SCR controls.

## Discussion

Genetic variants in the X-linked *MECP2* gene are most commonly associated with RTT, a devastating neurodevelopmental disorder that predominantly affects females. However, *MECP2* variants have also been identified in patients with other neurodevelopmental conditions, including Angelman-like syndrome and Attention-Deficit Hyperactivity Disorder, and more rarely in autism spectrum disorders (9). These mutations generally result in partial or complete loss of MeCP2 function, indicating that maintaining sufficient levels of MeCP2 is essential for proper brain development and function. Conversely, increased *MECP2* gene dosage causes the MECP2 duplication syndrome, a severe neurodevelopmental disorder that predominantly affects males (34). Collectively, this genetic evidence highlights *MECP2* as a prototypical “Goldilocks” gene, whose expression must be tightly regulated, as both insufficient and excessive levels are detrimental.

The identification of disease-modifying therapies for RTT has likely been hampered by the functional versatility of MeCP2, together with its high abundance and stringent dosage control in the brain, where it exerts its primary functions. Indeed, MeCP2 deficiency is known to cause subtle yet consistent perturbations across multiple molecular pathways, making it unlikely that treatment with a single small molecule will confer substantial therapeutic benefit. Rather, RTT patients are more likely to benefit from combinatorial strategies targeting multiple pathways.

An alternative approach has emerged from advances in molecular biology, including gene therapy and genome or RNA editing. Accordingly, two gene therapy-based clinical trials for RTT are currently ongoing, with preliminary reports from the sponsoring companies appearing encouraging. Nevertheless, concerns related to precise control of MeCP2 dosage, together with the high costs associated with these interventions, underscore the importance of developing complementary molecular therapies that directly target the *MECP2* gene or its transcripts.

The use of ACE-tRNAs to promote readthrough of PTCs encoded in *MECP2* represents a valuable precision medicine strategy worthy of further investigation. Indeed, nearly 35% of RTT cases are caused by *MECP2* nonsense mutations, and four of the eight most prevalent disease-causing variants correspond to premature termination codons (10). Moreover, because readthrough-based therapies, including ACE-tRNAs, are agnostic to the specific gene harboring the PTC, and because nonsense mutations underlie more than 1,000 severe genetic disorders (35), a single therapeutic formulation of ACE-tRNAs has the potential to be used in a broad range of diseases. This feature may substantially reduce development and implementation costs, an aspect that is particularly relevant for rare disorders. In addition, compared with chemical readthrough agents, which frequently induce amino acid misincorporation co-translationally, ACE-tRNA–mediated suppression does not introduce missense substitutions and displays minimal readthrough of natural termination codons (NTCs, 36). Another clinically relevant feature is the small size of the ACE-tRNA transgene (~150 bp), which offers flexibility in viral and non-viral delivery strategies (27, 28, 36, 37). This compact genetic cargo also allows the inclusion of multiple cassettes of ACE-tRNAs within a single vector to enhance readthrough efficiency (23, 37).

Importantly, in the context of *MECP2* PTCs, readthrough therapies preserve the physiological regulation of gene expression by acting post-transcriptionally. Moreover, unlike conventional gene complementation strategies, ACE-tRNAs cannot induce “super rescue” of MeCP2 expression, even in cells from RTT heterozygous patients that express the wild-type allele, thereby minimizing possible toxicity stemming from MeCP2 overexpression.

Although only a limited number of disease contexts have been tested thus far, sup-tRNA technologies have been shown to efficiently suppress nonsense mutations in their native genomic context, both for *CFTR* in cultured human cells and for *Idua* (α-L-iduronidase) in a mouse model of Hurler syndrome (22, 28, 29, 38). These considerations prompted us to investigate the efficacy of ACE-tRNAs on *MECP2* PTCs, using a reductionist experimental system based on transient expression of common nonsense variants. We focused on three pathogenic arginine CGA to UGA PTCs, as this class of nonsense mutation is highly prevalent in RTT, accounting for approximately 30% of RTT cases (10).

Our data demonstrate robust ACE-tRNA-mediated readthrough across all tested variants. When readthrough efficiency was quantified by comparing rescued full-length MeCP2 to wild-type protein levels, R270X showed the highest efficiency (>85%), whereas R255X displayed the lowest (>57%). When efficiency was instead calculated as the proportion of full-length protein relative to total MeCP2 products (including truncated species), R168X exhibited the highest readthrough of 64% of WT, while R255X again showed the lowest efficiency at 47% of WT. The varied level of PTC suppression observed across the MeCP2 variants may reflect the accumulation of truncated protein species that escaped suppression or may be influenced by the local nucleotide context surrounding each PTC, which is known to influence suppression efficiency (39, 40).

Indeed, differences in DNA-dose-dependent PTC suppression using this arginine ACE-tRNA have previously been reported for several different *CFTR* cDNA PTC variants (22).

In a previous study using plasmid DNA expressing 4X arginine ACE-tRNA, PTC suppression did not reach saturation even at the highest DNA dose transfected in HEK293T cells (22). This likely reflects the translational dynamics associated with the arginine ACE-tRNA, including transcription and processing of the ACE-tRNA, amino acid charging by the arginyl-tRNA synthetase, delivery to the ribosome by EF1a, and effective decoding at the ribosome (22). Further, the pool of arginyl-ACE-tRNA competes with the translation termination release factors (eRF1 and eRF3), which generally results in some amount of termination even when using highly efficient ACE-tRNAs. Indeed, near-WT rescue of a PTC-containing protein has been demonstrated (37), however this required highly efficient delivery of a DNA minivector encoding a highly active leucine ACE-tRNA targeting a genomically encoded PTC in a *CFTR* transcript that is expressed at low levels.

This discrepancy likely reflects the accumulation of truncated protein species that did not undergo suppression or the influence of PTC nucleotide context.

By analyzing readthrough efficiency as a function of dose, we further demonstrated, consistent with previous studies (26, 36), that all tested PTCs benefit from vectors containing multiple ACE-tRNA expression cassettes, although certain mutations, exemplified here by R255X, are recoded less efficiently. While PTCs located near the NTC might be expected to resist readthrough due to evolutionary pressure favoring efficient translation termination at NTCs (22), this feature alone cannot explain our results, particularly considering that the efficiently recoded R270X variant occurs at a more downstream position within the coding sequence.

Two factors are therefore likely to influence readthrough efficiency: the nucleotide sequence context flanking the PTC (22, 23, 36) and the local ribosomal translation speed within approximately 35 codons upstream of the PTC. Indeed, abrupt changes in ribosomal elongation rate in this region have been shown to reduce ACE-tRNA–mediated suppression by increasing the probability of ribosomal collisions (41). Although these findings require validation in a genomic context, they suggest that inter-allelic heterogeneity in therapeutic response may exist, depending on the specific mutation carried by the individual.

Finally, we demonstrated that ACE-tRNA rescued MeCP2 correctly localizes to highly methylated pericentromeric heterochromatin in murine cells and recruits TBL1, a core subunit of the NCoR/SMRT transcriptional corepressor complexes. To our knowledge, these features have not previously been jointly assessed in preclinical readthrough studies targeting *MECP2* nonsense mutations and together provide strong evidence for functional recovery. Immunofluorescence analyses revealed that virtually all cells co-transfected with the R168X variant and the corresponding ACE-tRNA vector regained MeCP2 localization at heterochromatic foci, indicating efficient readthrough across the cell population. In triple co-transfection experiments (performed with all 3 nonsense mutations), recoded MeCP2 and TBL1 consistently co-localized at heterochromatic foci, although variable amounts of TBL1 remained cytoplasmic in some cells, likely reflecting limiting MeCP2 expression relative to TBL1.

The most notable concern associated with readthrough technologies is the potential off-target suppression of natural termination codons (NTCs), leading to C-terminally extended proteins that could result in toxicity. In 2019, Lueck et al., performed ribosomal profiling in cultured mammalian cells transfected with plasmid DNA encoding highly active ACE-tRNAs and found that transcriptome-wide NTC readthrough was not appreciable under these conditions (20). Several studies have corroborated these findings following delivery of sup-tRNAs by AAV (29), as RNA using lipid nanoparticles (28) or by transfection of sup-tRNA plasmid DNA (42). Together, these results support the idea that NTCs have evolved to be inherently less susceptible to suppression than PTCs, an idea that has been discussed in depth elsewhere (21, 36, 39, 40). Nonetheless, the long-term off-target and tissue-specific safety of ACE-tRNAs, particularly under chronic administration, remains to be fully established.

In conclusion, this study demonstrates high efficiency of ACE-tRNA–mediated readthrough of CGA-to-UGA *MECP2* nonsense mutations and significant functional rescue of MeCP2 protein. These results represent an initial step of a longer path aimed at determining whether the levels of recoded MeCP2 achieved are sufficient to exceed the therapeutic threshold required to reverse the effects of pathogenic *MECP2* PTCs. In this context, even modest restoration of full-length MeCP2 may be sufficient to confer meaningful functional benefits, suggesting that complete normalization of protein levels may not be necessary to achieve therapeutic efficacy (43). It will also be important to assess whether this threshold can be reached across all CGA to UGA variants of *MECP2* and ultrarare nonsense mutations. To this end, our future studies will be conducted in a genomic context, ideally in human cells and *in vivo*, using available disease-relevant mouse models.

## Data Availability

The raw data supporting the conclusions of this article will be made available by the authors, without undue reservation.

## Supplementary material

SUPPLEMENTARY FIGURE S1 ACE-tRNA mediated readthrough does not affect the MeCP2 nucleoplasmic-to-total nuclear signal ratio. The graph shows the nucleoplasmic-to-total nuclear signal ratio (%). Each dot represents a cell from three coverslips derived from independent experiments (biological *n* = 3). Data are presented as mean ± SEM, ^****^*p* < 0.0001; Kruskal-Wallis test followed by Dunn’s multiple comparisons test.
