# Supplementary figures and images for: Anticodon-engineered tRNAs restore full-length MeCP2 expression and function in Rett syndrome nonsense mutations
Source: Front Neurol. 2026 Jun 1;17:1778877. doi: 10.3389/fneur.2026.1778877 (PMC13267816; doi:10.3389/fneur.2026.1778877)

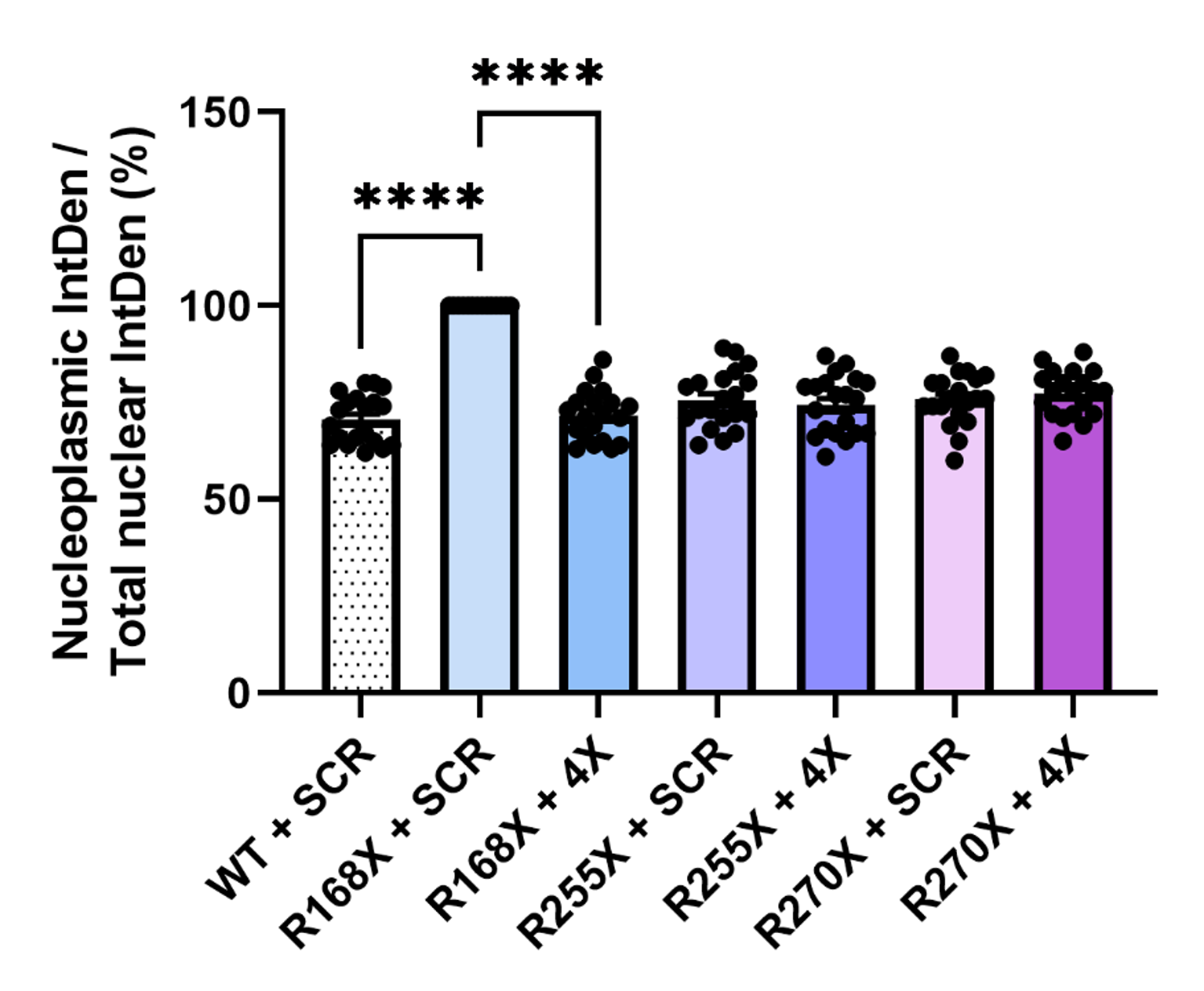

Supplement: SUPPLEMENTARY FIGURE S1 — ACE-tRNA mediated readthrough does not affect the MeCP2 nucleoplasmic-to-total nuclear signal ratio. The graph shows the nucleoplasmic-to-total nuclear signal ratio (%). Each dot represents a cell from three coverslips derived from independent experiments (biological n = 3). Data are presented as mean ± SEM, ****p < 0.0001; Kruskal-Wallis test followed by Dunn’s multiple comparisons test. [file Image_1.TIF]
